# In vitro IgE diagnostics in inhalant allergy: Plant and mold allergens 

**DOI:** 10.5414/ALX02616E

**Published:** 2026-07-01

**Authors:** Regina Treudler

**Affiliations:** Institute of Allergology, Charité – Universitätsmedizin Berlin, Corporate Member of Freie Universität Berlin and Humboldt-Universität zu Berlin, Berlin, Germany

**Keywords:** allergy, inhalant, component resolved, diagnostic, review

## Abstract

Respiratory allergies represent one of the most prevalent immune-mediated disorders worldwide, such as allergic rhinitis and asthma. The advent of in vitro diagnostic methods, particularly those based on molecular allergology, has revolutionized the diagnostic approach to inhalant allergies by enabling precise identification of sensitizing allergens at the molecular level. This review presents an analysis of the current status of in vitro diagnostics in respiratory allergy to plants and molds, with emphasis on molecular diagnostics for key allergens from trees (e.g., birch/*Betula verrucosa*), grasses (Poaceae family), weeds (e.g.mugwort/*Artemisia vulgaris*, ragweed/*Ambrosia artemisiifolia*), and molds (e.g. *Alternaria, Aspergillus).* We discuss major allergenic proteins, diagnostic tools, implications for precision medicine, and integration with precision immunotherapy.

## Introduction 

**Figure 1:**
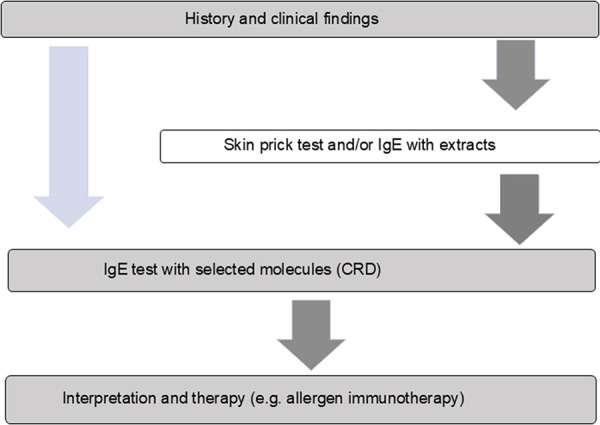
Diagnostic work-up in inhalant allergy to plants. While standard work up includes skin prick test and specific IgE with extracts, it may be worth performing complement resolved diagnostics (CRD) from the outset if allergen immunotherapy is considered.

Allergic diseases have emerged as a major health concern over the past decades, with inhalant allergens being the most common triggers of respiratory allergies. Prevalence rates vary regionally but are estimated to affect 20 – 30% of the global population [26]. In Germany, within a representative cohort investigation, the prevalence of bronchial asthma was 8.6% and that of hay fever was 14.8%. IgE sensitization to inhalant allergens was found in 33.6% [2] . Inhalant plant allergens include pollens and molds, which can induce a type I hypersensitivity reaction mediated by immunoglobulin E (IgE). Most relevant plant allergens in Germany are birch, grass, and – to a lesser extent – mugwort and other weeds. Climate change is leading to longer seasons of exposure to airborne pollen and spores. In addition, allergens from neophytes (ragweed, tree of heaven, and others) are becoming increasingly clinically significant [19, 25]. The mold species with greatest clinical relevance are *Alternaria alternata* (outdoor) and *Aspergillus fumigatus* (indoor) [6]. Traditional diagnostic approaches, such as skin prick testing (SPT) and serum specific IgE detection against whole allergen extracts, provide valuable insights but often lack specificity due to cross-reactivity between homologous proteins [6, 23, 24]. SPT and specific IgE are two tests that are not always concordant, and show different sensitivity and specificity distinct for each allergen. In clinical practice, both tests should be used depending on clinical history features and obtained findings. During the last years, specific IgE diagnostics using whole allergen extracts has been increasingly supplemented and, in some cases, replaced by molecular diagnostics, also known as component-resolved diagnostics (CRD) [23]. CRD allows to identify IgE sensitization profiles at the level of individual allergen molecules [6, 15, 21, 23]. It may be worth performing CRD from the outset if allergen immunotherapy (AIT) is considered (Figure 1). 

This paper aims to provide an overview of the molecular in vitro diagnostic landscape for major inhalant allergens, with emphasis on clinical utility, molecular allergen characterization, and the role of CRD in guiding diagnosis and therapy. Component-resolved analysis is advisable when selecting candidates for pollen or mold immunotherapy [26]. 

## Molecular basis of inhalant allergy 

### IgE-mediated hypersensitivity 

The pathophysiology of inhalant allergy involves the production of allergen-specific IgE antibodies in predisposed individuals. Upon re-exposure, allergens cross-link IgE bound to FcεRI receptors on mast cells and basophils, leading to degranulation and release of histamine, leukotrienes, and cytokines. This results in the clinical manifestations of allergic rhinitis, conjunctivitis, or asthma [1, 24]. 

### Crossreactivity and pan-allergens 

**Figure 2:**
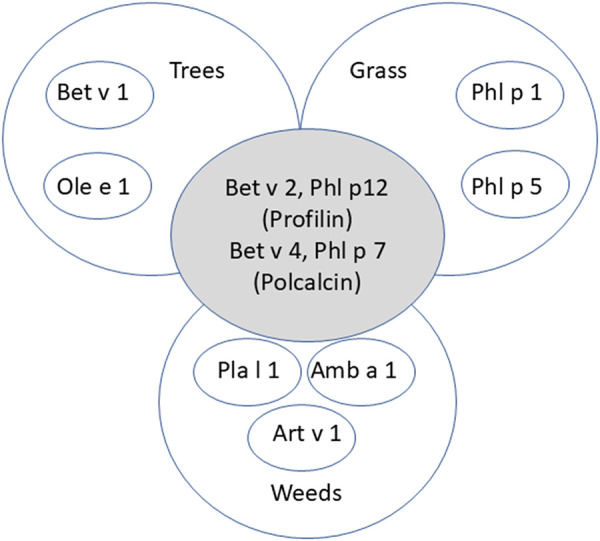
Trees, grasses, and weeds share common pan-allergens (i.e., profilins and polcalins), which can lead to false positive reactions in skin prick test or whole allergen extracts. Examples for major allergens are given, see also Tables 1 and Table 2.

A major challenge in allergy diagnosis is cross-reactivity due to homologous proteins across species. Pan-allergens such as profilins, polcalcins, and pathogenesis-related proteins (e.g., PR-10 family) can lead to broad sensitization patterns, complicating diagnostic and therapeutic strategies (Figure 2) [6, 23]. Molecular diagnostics enables differentiation between genuine sensitization and cross-reactivity. In vitro cross-reactions might occur between profilins (Bet v 2, Phl p 7) and polcalcins (e.g. Bet v 4, Phl p 12, Art v 4), Cross-reactivity between pectate lyases (e.g. Amb a 1, Art v 6) was shown to be minimal [17]. 

Ole e 1 proteins (e.g., Ole e 1, Pla l 1) are taken as species-specific marker allergens for olive (*Oleaceae*) and plane tree pollen, respectively. However, Pla I 1 and Ole e 1, share common epitopes, which can be cross-recognized by different antibodies and sera from different patients [4, 9]. While lipid transfer proteins (LTPs) (e.g. Art v 3, Amb a 6,) are frequently cross-reactive with food allergen Pru p 3 from peach, cross-reactivity between pollen LTP seems to be rare [12]. 

Molecular diagnostics is particularly relevant in polysensitized patients, in distinguishing primary sensitization from cross-reactivity, and in selecting candidates for AIT [15]. 

## Major aeroallergens and their molecular diagnostics 

### Tree pollen 

The two most frequently implicated families in tree-pollen allergies are (i) Betulaceae including the genera* Alnus *(alder),* Betula *(birch),* Corylus *(hazel), and (ii)* Fagaceae, *comprising the genera* Fagus *(beech), and* Quercus *(oak) [6]. 

Other tree orders are Lamiales (e.g., ash/*fraxinus*, olive/*olea*), Pinales (e.g., cypress/*cupressus*) and Proteales (e.g., plane tree/*Platanus*), which are of minor relevance in central Europe [6]. Pathogenesis-related-protein group 10 (PR-10) molecules (i.e., Bet v 1 and homologous allergens) are the major allergens in Fagales pollen and are recognized by virtually all allergic patients. Tree pollens belong to the most important respiratory allergen sources, and birch allergy is of outmost importance in Central and Northern Europe, with alder and hazel showing strong cross-reactivity. Recently, pollen from tree of heaven (*Ailanthus*) were reported to gain relevance in Germany. However, so far, no routine diagnostic method is available for this allergen [18]. 

**Birch (*Betula verrucosa*), Alder (*Alnus glutinosa*), Hazel (*Corylus avellana*)**

Bet v 1 is the primary marker of genuine birch pollen sensitization [6]. Sensitization to Bet v 2 and Bet v 4 usually indicate cross-reactivity, e.g., with grass, mugwort, or ragweed pollen. Molecular IgE testing against Bet v 1 allows differentiation between primary birch allergy and cross-reactive pollen–food syndrome [6]. Bet v 7 (cyclophilin) was recently identified as another neglected panallergen [14]. Major allergens Aln g 1 and Cor a 1 belong to the PR10 family and show high cross-reactivity to Bet v 1 [6]. 

**Oak (*Quercus acutissima*), Beech, Ash (*Fraxinus excelsior*), Olive (*Olea europaea*)**

Allergens of these trees are of minor importance in patients with respiratory symptoms in Central Europe. However, diagnostics including specific IgE investigation may be performed in patients with seasonal symptoms in spring time/early summer. Major allergen from ash is highly cross-reactive with major olive allergen Ole e 1, which therefore can be chosen for molecular diagnostics in suspected ash allergy. 

### Grass pollen 

**Grass pollen (Poaceae family)**

Phl p 1 and Phl p 5 (timothy grass) are recognized by > 90% of grass-allergic patients [6]. IgE to Phl p 1 and Phl p 5 indicates primary grass sensitization. Sensitization to Phl p 7 and Phl p 12 suggests cross-reactivity [6] [Table Table1](Table 2). 

**Weed pollen**

Pollen of mugwort and – as a neophyte – ragweed are considered the main weed pollen allergy-eliciting sources in Central Europe. Pellitory and English plantain are of lower clinical relevance in Central compared with Southern Europe (Table 2). 

**Mugwort (*Artemisia vulgaris*)**

Art v 1 is a marker for genuine mugwort sensitization [7], while Art v 3 is clinically relevant due to its stability and association with severe reactions, particularly food-related reactions (e.g., Pru p 3 from peach) [8]. CRD helps distinguish primary mugwort allergy from cross-reactivity with other pollens or LTP-containing foods. Mugwort allergens show strong cross-reactions with ragweed pollen; Art v 1 and Amb a 4 as well as Art v 6 and Amb a 1 are strongly related [6]. 

**Ragweed (*Ambrosia artemisiifolia*)**

Amb a 1 is the key diagnostic marker for ragweed allergy. [5]. Profilin and polcalcin reactivities indicate cross-sensitization. Amb a 1-based diagnostics support targeted immunotherapy decisions [6]. 

**Molds**

The clinical availability of both SPTs and serological tools for IgE-mediated mold allergy diagnosis has been continuously decreasing, and standardization of mold extract is still difficult [10]. 

The concordance between skin tests and serological tests can be less than 30%, depending on the mold species. 

***Alternaria alternata***

Alt a 1 (Table 2) is highly specific for *Alternaria* allergy, making it a reliable diagnostic marker [6, 20]. Molecular diagnosis distinguishes *Alternaria* sensitization from cross-reactivity with other molds, which is clinically relevant in asthma [3]. 

**Aspergillus fumigatus**

Among clinically important *A. fumigatus* single allergens, Asp f 1 is a major allergen in patients suffering from allergic bronchopulmonary aspergillosis (ABPA) (80 – 85%) as well as in *A. fumigatus*-sensitized asthmatics (50 – 84.5%) [6]. IgE to Asp f 2, 4, and 6 (Table 2) are more frequently positive in patients with ABPA than in astmatics [6]. In patients with cystic fibrosis or ABPA, Asp f 4 is a major allergen [6]. 

## Clinical implications of molecular diagnostics 

A notable advantage of molecular diagnostics is its ability to reduce misinterpretation caused by false positive reactions in SPTs. False positives can arise from non-specific skin reactivity, cross-reactivity among pan-allergens, or poor standardization of allergen extracts. By using purified allergen molecules, molecular diagnostics allows clinicians to distinguish between true sensitization and cross-reactivity, thereby improving specificity. This is particularly relevant in polysensitized patients, where SPT may yield multiple positive results that do not correlate with clinical symptoms. Molecular diagnostics therefore enhances diagnostic accuracy, prevents over-diagnosis, and avoids unnecessary avoidance strategies or inappropriate immunotherapy [23, 28]. 

Moreover, the use of molecular diagnostics in AIT shows the following advantages [11, 13]:

- Molecular marker allergens help to identify clinically relevant inhalant allergen sources that are candidates for molecular allergy diagnostics to identify specific allergen sensitivities (Tables 1, Table 2).
- Molecular marker allergens for allergen-specific IgE detection help to separate specific sensitizations to tree and/or grass and/or weed pollen from IgE sensitization to pollen panallergens.
- Allergen extract compositions based on confirmed IgE sensitizations to major allergen markers might facilitate more targeted allergen-specific immunotherapy.

AIT with major allergens, i.e., Bet v 1, has been applied in clinical trials [22], but no such allergen extracts with major allergens are commercially available yet. 

Overall, it will not always be possible to choose a tailored AIT extract for each patient. Five categories of molecular matching/mismatching between the IgE sensitization profile of the patient and the molecular composition of an AIT preparation have been described [13]:

- Perfect matching – The molecular composition of the AIT corresponds exactly to the patient’s IgE molecular sensitization profile.
- Underpowered immunization – Some patients were sensitized to more allergenic molecules than contained in the immunotherapy. In this case, the efficacy of the immunization might be insufficient.
- Overpowered immunization – The immunotherapy preparation contains more molecules than the individual patient’s sensitization profile. Three consequences could result: a beneficial induction of IgG antibodies and/or prevention of new IgE sensitization, an unwanted IgE sensitization to these molecules, and no effect.
- Underpowered/overpowered immunization – This category includes patients with a mixed match/mismatch. Also, the expected effects of such a mismatch might be any of the previously mentioned options.
- Unrelated immunization – In this case, the immunization does not fit the sensitization profile of the patient. This category bears the lowest (if any) expected benefit, as well as the highest risk of unwanted effects

## Conclusion and unmet needs 

Molecular diagnostics have transformed the field of inhalant allergy by enabling precise, component-resolved evaluation of allergen sensitization. For key allergens such as birch, grasses, mugwort, ragweed, and *Alternaria*, molecular markers allow differentiation between genuine sensitization and cross-reactivity, guiding both diagnosis and immunotherapy decisions. Future developments should further integrate CRD with precision medicine approaches, improving the management of allergic diseases. For this purpose it would be desirable for a greater number of component-resolved allergens to be available. This would decrease the number of non-unambiguous results when using whole allergen extracts in diagnosis of inhalant plant allergy. 

## Funding 

None. 

## Conflict of interest 

None. 

**Table 1. Table1:** Major and selected minor allergens in trees. Component-resolved diagnostics may be helpful in distinguishing unspecific cross-reactivity when using whole allergen extracts [6, 23, 27].

**Plant**	**Major allergen ** **(protein family/ function)**	**Selected other allergens ** **(protein family/ function)**
Birch *(Betula verruccosa)*	**Bet v 1** (PR10)	**Bet v 2** (profilin) **Bet v 4** (polcalcin) **Bet v 6** (ether reductase) Bet v 7 (cyclophilin)
Alder *(Alnus glutinosa)*	Aln g 1 (PR 10)	Aln g 4 (polcalcin)
Hazel *(Corylus avellana)*	**Cor a 1** (PR 10)	Cor a 2 (profilin)
Oak (*Quercus Acutissima*)	Que ac 1 (PR10)	Que ac 2 (profilin) Que ac 4 (polcalcin)
Beech *(Fagus sylvatica)*	Fag s 1 (PR10)	Fag s 2 (profilin) Fag s 4 (polcalcin)
Ash (*Fraxinus excelsior*)	Fra e 1 (olive group)	
Olive *(Olea europaea)*	**Ole e 1** (common olive group 1)	Ole e 2 (profilin) Ole e 3 (polcalcin) **Ole e 7** (LTP) **Ole e 9 (**1,3-β-glucanase)
Cypress (*Cupressus arizonica)*	**Cup a 1** (pectate lyase)	
Plane *(Platanus acerifolia)*	**Pla a 1** (invertase inhibitor)	**Pla a 3** (LTP)

PR10 = pathogenesis-related protein 10; LTP = lipid transfer protein. Marked in bold: commercially available single allergens.

**Table 2.** Major and selected minor allergens in grasses, weeds, and molds. Component-resolved diagnostics may be helpful in distinguishing unspecific cross-reactivity when using whole allergen extracts [6, 23, 27].

**Plant**	**Major allergen ** **(protein family/ function)**	**Selected other allergens ** **(protein family/ function)**
Grass		
Grass (*Phleum pretense, Poaceae family)*	**Phl p 1** (CCD bearing protein) **Phl p 5** (grass group 5)	Phl p 7 (polcalcin) Phl p 12 (profilin)
Weeds		
Mugwort *(Artemisia vulgaris)*	**Art v 1** (defensin-like protein) Art v 3 (LTP).	Art v 4 (profilin) Art v 5 (polcalcin)
Ragweed (*Ambrosia artemisiifolia*)	**Amb a 1** (pectate lyase)	Amb a 4 (defensin-like protein) Amb a 6 (LTP) Amb a 8 (profilin) Amb a 9/10 (polcalcins)
Pellitory *(Parietaria judaica)*	**Par j 2** (LTP)	Par j 1 (LTP) Par j 3 (profilin) Par j 4 (polcalcin)
English plantain (*Plantago lanceolata*)	**Pla l 1 (**Ole e 1 like protein)	Pla l 2 (profilin)
Molds		
*Alternaria alternata*	**Alt a 1** (glycoprotein)	Alt a 6 (enolase)
*Aspergillus fumigatus*	**Asp f 1** (mitogillin family) **Asp f 2** (metalloprotease) **Asp f 3** (peroxysomal protein) **Asp f 4** (unknown function)	**Asp f 6** (mangan superoxide dismutase)

PR10 = pathogenesis-related protein 10; LTP = lipid transfer protein. Marked in bold: commercially available single array allergens

## References

[1] Averbeck M Gebhardt C Emmrich F Treudler R Simon JC Immunologic principles of allergic disease. J Dtsch Dermatol Ges. 2007; 5: 1015–1028. 17976144 10.1111/j.1610-0387.2007.06538.x

[2] Bergmann K-C Heinrich J Niemann H Current status of allergy prevalence in Germany: Position paper of the Environmental Medicine Commission of the Robert Koch Institute. Allergo J Int. 2016; 25: 6–10. 27069844 10.1007/s40629-016-0092-6 PMC4792334

[3] Bush RK Prochnau JJ Alternaria-induced asthma. J Allergy Clin Immunol. 2004; 113: 227–234. 14767434 10.1016/j.jaci.2003.11.023

[4] Castro AJ Alché JD Calabozo B Rodríguez-García MI Polo F Pla 1 1 and Ole e 1 pollen allergens share common epitopes and similar ultrastructural localization. J Investig Allergol Clin Immunol. 2007; 17: 41–47. 18050571

[5] Cheng Z-L Ma T-T Gao Z-S Ming W-H Yang M-R Wang X-Y Global Ragweed Allergy: Molecular Allergens and Integrated Control Strategies. J Asthma Allergy. 2025; 18: 403–416. 40099306 10.2147/JAA.S506897 PMC11911648

[6] Dramburg S Hilger C Santos AF de Las Vecillas L Aalberse RC Acevedo N Aglas L Altmann F Arruda KL Asero R Ballmer-Weber B Barber D Beyer K Biedermann T Bilo MB Blank S Bosshard PP Breiteneder H Brough HA Bublin M. EAACI Molecular Allergology User’s Guide 2.0. Pediatr Allergy Immunol. 2023; 34: e13854. 37186333 10.1111/pai.13854

[7] Gadermaier G Jahn-Schmid B Vogel L Egger M Himly M Briza P Ebner C Vieths S Bohle B Ferreira F Targeting the cysteine-stabilized fold of Art v 1 for immunotherapy of Artemisia pollen allergy. Mol Immunol. 2010; 47: 1292–1298. 20022115 10.1016/j.molimm.2009.11.029

[8] Gadermaier G Wopfner N Wallner M Egger M Didierlaurent A Regl G Aberger F Lang R Ferreira F Hawranek T Array-based profiling of ragweed and mugwort pollen allergens. Allergy. 2008; 63: 1543–1549. 18925891 10.1111/j.1398-9995.2008.01780.x

[9] Hauser M Roulias A Ferreira F Egger M Panallergens and their impact on the allergic patient. Allergy Asthma Clin Immunol. 2010; 6: 1. 20298513 10.1186/1710-1492-6-1 PMC2830198

[10] Hurraß J Heinzow B Walser-Reichenbach S Aurbach U Becker S Bellmann R Bergmann K-C Cornely OA Engelhart S Fischer G Gabrio T Herr CEW Joest M Karagiannidis C Klimek L Köberle M Kolk A Lichtnecker H Lob-Corzilius T Mülleneisen N AWMF mold guideline “Medical clinical diagnostics for indoor mold exposure” – Update 2023 AWMF Register No. 161/001. Allergol Select. 2024; 8: 90–198. 38756207 10.5414/ALX02444E PMC11097193

[11] Kleine-Tebbe J Matricardi PM Hamilton RG Allergy Work-Up Including Component-Resolved Diagnosis: How to Make Allergen-Specific Immunotherapy More Specific. Immunol Allergy Clin North Am. 2016; 36: 191–203. 26617235 10.1016/j.iac.2015.08.012

[12] Lombardero M García-Sellés FJ Polo F Jimeno L Chamorro MJ García-Casado G Sánchez-Monge R Díaz-Perales A Salcedo G Barber D Prevalence of sensitization to Artemisia allergens Art v 1, Art v 3 and Art v 60 kDa. Cross-reactivity among Art v 3 and other relevant lipid-transfer protein allergens. Clin Exp Allergy. 2004; 34: 1415–1421. 15347375 10.1111/j.1365-2222.2004.02053.x

[13] Matricardi PM Dramburg S Potapova E Skevaki C Renz H Molecular diagnosis for allergen immunotherapy. J Allergy Clin Immunol. 2019; 143: 831–843. 30850070 10.1016/j.jaci.2018.12.1021

[14] Matricardi PM Potapova E Panetta V Lidholm J Mattsson L Scala E Bernardini R Caffarelli C Casani A Cervone R Chini L Comberiati P De Castro G Miraglia Del Giudice M Dello Iacono I Di Rienzo Businco A Gallucci M Giannetti A Moschese V Varin E IgE to cyclophilins in pollen-allergic children: Epidemiologic, clinical, and diagnostic relevance of a neglected panallergen. J Allergy Clin Immunol. 2024; 153: 1586–1596.e2. 38513837 10.1016/j.jaci.2024.01.030

[15] Matricardi PM van Hage M Custovic A Korosec P Santos AF Valenta R Molecular allergy diagnosis enabling personalized medicine. J Allergy Clin Immunol. 2025; 156: 485–502. 39855360 10.1016/j.jaci.2025.01.014

[16] Pawankar R Canonica GW Holgate ST Lockey RF Allergic diseases and asthma: a major global health concern. Curr Opin Allergy Clin Immunol. 2012; 12: 39–41. 22157151 10.1097/ACI.0b013e32834ec13b

[17] Pichler U Hauser M Wolf M Bernardi ML Gadermaier G Weiss R Ebner C Yokoi H Takai T Didierlaurent A Rafaiani C Briza P Mari A Behrendt H Wallner M Ferreira F Pectate lyase pollen allergens: sensitization profiles and cross-reactivity pattern. PLoS One. 2015; 10: e0120038. 25978036 10.1371/journal.pone.0120038 PMC4433284

[18] Prenzel F Treudler R Lipek T Vom Hove M Kage P Kuhs S Kaiser T Bastl M Bumberger J Genuneit J Hornick T Klotz S Zarnowski J Boege M Zebralla V Simon J-C Dunker S Invasive Growth of Ailanthus altissima Trees is Associated with a High Rate of Sensitization in Atopic Patients. J Asthma Allergy. 2022; 15: 1217–1226. 36071746 10.2147/JAA.S373177 PMC9443999

[19] Röseler STM Baron JM Höflich C Merk HF Bas M Bier H Dott W Fietkau K Hajdu Z Kaiser L Kraus T Laven G Moll-Slodowy S Mücke H-G Straff W Wurpts G Yazdi AS Chaker A Balakirski G “New” inhalant plant allergens. Allergol Select. 2020; 4: 1–10. 32357199 10.5414/ALX02066E PMC7189803

[20] Torres-Borrego J Suárez-Pérez J Aliaga-Mazas Y Burgos AM-C Nevot-Falcó S Allergy to Alternaria alternata: Comprehensive review from the origin to the therapeutic approach. Allergol Immunopathol (Madr). 2025; 53: 179–188. 40923435 10.15586/aei.v53i5.1454

[21] Treudler R Update on in vitro allergy diagnostics J Dtsch Dermatol Ges. 2012; 10: 89–97. 22233232 10.1111/j.1610-0387.2011.07860.x

[22] Treudler R Franke A Schmiedeknecht A Ballmer-Weber B Worm M Werfel T Jappe U Biedermann T Schmitt J Brehler R Kleinheinz A Kleine-Tebbe J Brüning H Ruëff F Ring J Saloga J Schäkel K Holzhauser T Vieths S Simon JC BASALIT trial: double-blind placebo-controlled allergen immunotherapy with rBet v 1-FV in birch-related soya allergy. Allergy. 2017; 72: 1243–1253. 27998002 10.1111/all.13112

[23] Treudler R Simon JC Overview of component resolved diagnostics. Curr Allergy Asthma Rep. 2013; 13: 110–117. 23076421 10.1007/s11882-012-0318-8

[24] Treudler R Simon J-C Update on Type-1 Allergy Diagnostics. Handb Exp Pharmacol. 2022; 268: 393–403. 34173866 10.1007/164_2021_487

[25] Treudler R Simon J-C Developments and perspectives in allergology J Dtsch Dermatol Ges. 2023; 21: 399–403. 10.1111/ddg.15034 37070510

[26] Tripodi S Frediani T Lucarelli S Macrì F Pingitore G Di Rienzo Businco A Dondi A Pansa P Ragusa G Asero R Faggian D Plebani M Matricardi PM Molecular profiles of IgE to Phleum pratense in children with grass pollen allergy: implications for specific immunotherapy. J Allergy Clin Immunol. 2012; 129: 834–839. 22206774 10.1016/j.jaci.2011.10.045

[27] WHO/IUIS Allergen Nomenclature Sub-Committee. Allergen Nomenclature. https://allergen.org/search.php.

[28] Zemelka-Wiacek M Agache I Akdis CA Akdis M Casale TB Dramburg S Jahnz-Różyk K Kosowska A Matricardi PM Pfaar O Shamji MH Jutel M Hot topics in allergen immunotherapy, 2023: Current status and future perspective. Allergy. 2024; 79: 823–842. 37984449 10.1111/all.15945

